# Connexin Signaling in the Juxtaglomerular Apparatus (JGA) of Developing, Postnatal Healthy and Nephrotic Human Kidneys

**DOI:** 10.3390/ijms21218349

**Published:** 2020-11-06

**Authors:** Ivona Kosovic, Natalija Filipovic, Benjamin Benzon, Ivana Bocina, Merica Glavina Durdov, Katarina Vukojevic, Marijan Saraga, Mirna Saraga-Babic

**Affiliations:** 1 Department of Anatomy, Histology and Embryology, School of Medicine, University of Split, 21000 Split, Croatia; ikosovic@mefst.hr (I.K.); natalija.filipovic@mefst.hr (N.F.); benzon.benjamin@gmail.com (B.B.); kvukojev@gmail.com (K.V.); 2 Department of Biology, Faculty of Science, University of Split, 21000 Split, Croatia; bocina@pmfst.hr; 3 Department of Pathology, University Hospital in Split, School of Medicine, University of Split, 21000 Split, Croatia; merigdst@yahoo.co.uk; 4 Department of Paediatrics, University Hospital in Split, School of Medicine, University of Split, 21000 Split, Croatia; msaraga@kbsplit.hr

**Keywords:** kidney development, CNF, connexins, renin, blood pressure control

## Abstract

Our study analyzed the expression pattern of different connexins (Cxs) and renin positive cells in the juxtaglomerular apparatus (JGA) of developing, postnatal healthy human kidneys and in nephrotic syndrome of the Finnish type (CNF), by using double immunofluorescence, electron microscopy and statistical measuring. The JGA contained several cell types connected by Cxs, and consisting of macula densa, extraglomerular mesangium (EM) and juxtaglomerular cells (JC), which release renin involved in renin-angiotensin- aldosteron system (RAS) of arterial blood pressure control. During JGA development, strong Cx40 expression gradually decreased, while expression of Cx37, Cx43 and Cx45 increased, postnatally showing more equalized expression patterning. In parallel, initially dispersed renin cells localized to JGA, and greatly increased expression in postnatal kidneys. In CNF kidneys, increased levels of Cx43, Cx37 and Cx45 co-localized with accumulations of renin cells in JGA. Additionally, they reappeared in extraglomerular mesangial cells, indicating association between return to embryonic Cxs patterning and pathologically changed kidney tissue. Based on the described Cxs and renin expression patterning, we suggest involvement of Cx40 primarily in the formation of JGA in developing kidneys, while Cx37, Cx43 and Cx45 might participate in JGA signal transfer important for postnatal maintenance of kidney function and blood pressure control.

## 1. Introduction

Kidney differentiation requires the genetically regulated interactions between the ureteric bud and the metanephric mesenchyme, subsequently leading to changes in mesenchymal cells known as mesenchymal to epithelial transition (MET) [1]. During this process, the metanephric mesenchyme first transforms into metanephric cup cells that give rise to renal vesicle, comma-shaped, and S-shaped body, followed by the capillary loop stages, ultimately resulting in the development of mature nephrons and glomeruli [2]. In order to achieve proper function, glomeruli must assemble appropriate circulation by the formation of renal arterioles [3]. Foxd1 stromal cells are believed to be progenitors not only for mesangial cells, smooth muscle cell, and interstitial pericytes but also for renin precursor cells. Recently, it has been speculated that pericytes might be the cells that are able to synthesize, store, and secrete renin in the human kidney [4,5]. During early development, renin- secreting cells appear in various tissues and organs, where they are engaged in important events such as vascular development, as well as in tissue repair and regeneration following injury [6,7]. Subsequently, the renin cells set into renal vasculature where they might play a role in proper vascular development, but afterward remain positioned only in the juxtaglomerular area. Thus, the juxtaglomerular cells (JC) are located in the wall of the afferent arteriole, at the entrance into the glomerulus [8,9]. They are part of the juxtaglomerular apparatus (JGA), a specialized sensory structure consisting also of a tubular component, the macula densa, and the extraglomerular mesangium (EM). Thus different cell types contribute to JGA, including vascular smooth muscle cells (VSMCs), endothelial cells, mesangial cells, macula densa cells, and renin-secreting JC [10,11]. Macula densa is composed of a group of 15–20 cells that perceive changes in tubular fluid composition and by feedback mechanisms regulate glomerular blood flow, and the rate of glomerular filtration. Renin release is considered to be dependent on the macula densa hypertonicity-induced shrinkage, while the inhibition is mainly in accordance with elevations of calcium concentration in JC [12]. Macula densa, via cell signaling, affects JG cells which take part in arteriolar vasoconstriction and renin release [12,13]. Upon reaching the circulation, renin, as the key regulating enzyme of renin- angiotensin- aldosterone system (RAS), causes the release of angiotensin II, a potent vasoconstrictor [14,15] which leads to elevated blood pressure. [6,13,16,17]. RAS plays a pivotal role in the regulation of blood pressure and fluid/electrolyte balance, thus regulating renal hemodynamics and function. However, the tissue RAS is also involved in other diverse physiological functions [18,19] and in various pathophysiological events, such as inflammation, vascular hypertrophy and thrombosis, but also hypertension and renal diseases [6,13,18,19,20,21,22]. Recent animal and clinical studies indicate an important role of RAS in the pathogenesis of acute kidney injury to chronic kidney disease transition (AKI-CKD) [23,24]. Increased renin release affecting an overactive RAS occur in the events of hypovolemia, sodium deprivation, low systemic blood pressure and sympathetic stimulation [13,16], while in conditions of threatened homeostasis, renin cells reestablish their plasticity resembling the one during development, and give rise to adjacent cells that can resume renin expression [4,6]. Interaction of the renin cells amongst themselves, and with other cells that form JGA is enabled by the cell to cell channels called the gap junctions. Connexins (Cxs) are integral membrane proteins which form gap junctions crucial for the intercellular signaling, and as such, are involved in many physiological and pathophysiological processes e.g., cell growth, proliferation and differentiation, homeostasis, development and tumorigenesis [10,25,26,27]. The regulation of renal function, particularly of JGA, depends on the direct cell-to-cell communications [11,28]. It has been described that disruptions of gap junctions may lead to modified preglomerular vascular tone and renin secretion [11]. Despite the former premise that macula densa lacks gap junctions, a study on rats had shown the presence of Cx37 in the basolateral membrane of macula densa cells [29]. Experimental studies led to speculations that arterial endothelial cells express Cx37 and Cx40, and possibly Cx43, whereas smooth muscle cells express Cx43 and Cx45. Renin-secreting cells seem to be characterized by Cx37, Cx40 and Cx45, but data vary in different studies [11,25,28,30,31]. It has been observed that Cx40 shows high expression in both the granular and the extraglomerular mesangial cells, while there are still discrepancies about Cx37 expression in those cells [32,33,34,35]. In experimental animals, changes in Cx expression have been associated with various pathological conditions such as diabetes and hypertension [28,36,37], and with conditions leading to chronic kidney disease (CKD) in mice models, showing increased Cx43 and decreased Cx37 [38]. Improvement of renal function has been shown in hypertensive mice with CKD which were treated with Cx43 antisense [39]. There is some evidence of connexin expression in adult human kidneys, mainly pointing the role of Cx43 in renal diseases [40,41]. Study on nephrectomy-obtained human tissues characterized the expression of Cx37, Cx40 and Cx43 in the JGA, with the highest abundance of Cx40 in that area, especially in renin-producing cells. In other study, Cx43 was highly increased in biopsies of patients with CKD [39].

Although there is not enough evidence about the interdependence between connexins and renin in human kidneys, especially during human development, data coming from experimental studies support the importance of this topic. Thus, the study on mice with deletion of the gene for Cx37 reported the relation of this connexin to the regulation of renin expression [42]. Cx40 is recognized as the predominant connexin of renin-producing cells in mice, although there are some inconsistencies in its role in fetal mice kidneys [34,43]. Based on a study on Cx40 –/– mice, Cx40 might be connected to renin release and renin-dependent hypertension [6,44]. Substitution of Cx40 with Cx45 in mice with high plasma renin and hypertension led to significantly reduced hypertension, thus pointing to role of Cx45 in blood pressure control [45,46]. That interesting finding implies that the role of other connexins in the juxtaglomerular apparatus, and their interchangeability, is yet to be addressed.

The understanding of renin and connexins signaling in human kidney, especially during development remains limited. Despite redundant investigations on gap junctions, specific localization and roles of different connexins in the JGA of fetal human kidney compared to the adult kidney needs to be clarified. The aim of our study was to describe patterning of Cxs and renin in JGA of developing and postnatal healthy human kidneys and nephrotic syndrome of the Finnish type (CNF) which gradually leads to CKD.

## 2. Results

The first signs of human glomeruli formation are observed in the 8th developmental week, when the s-shaped bodies embrace blood vessels to form immature glomeruli in the middle part of the developing kidney. In the 8th–10th week developing kidneys, expression of different Cxs, including Cx40, Cx43, Cx45 and Cx37 is observed throughout the kidney tubules and blood vessels, as well as in the JGA region, but with different expression pattern and intensity during time. Initial signs of JGA formation in the immature glomeruli are observed in the form of close association between the distal tubule (macula densa) and afferent arteriole as well as nearby mesenchyme (giving rise to extraglomerular mesangium) already in the earliest developmental stages. In order to analyze the progression of JGA formation in histological sections of developing, postnatal and nephrotic kidneys, we applied double immunofluorescence, semi-thin sections, statistical methods and electron microscopy.

### 2.1. Double Immunofluorescence Staining of Renin and Different Cxs in Developing and Postnatal JGA of Human Kidneys

#### 2.1.1. Cx40 Expression

**Figure 1:**
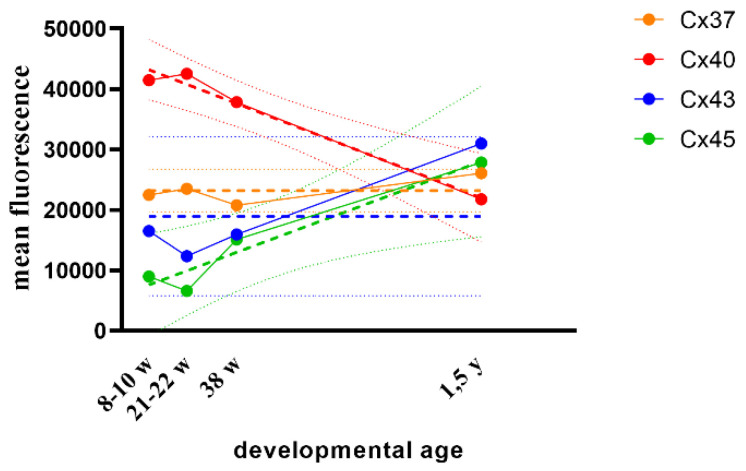
Scatter plot with error bars (95% CI) showing differences in the course of Cx37, Cx40, Cx43 and Cx45 expression during human kidney development.

**Figure 2:**
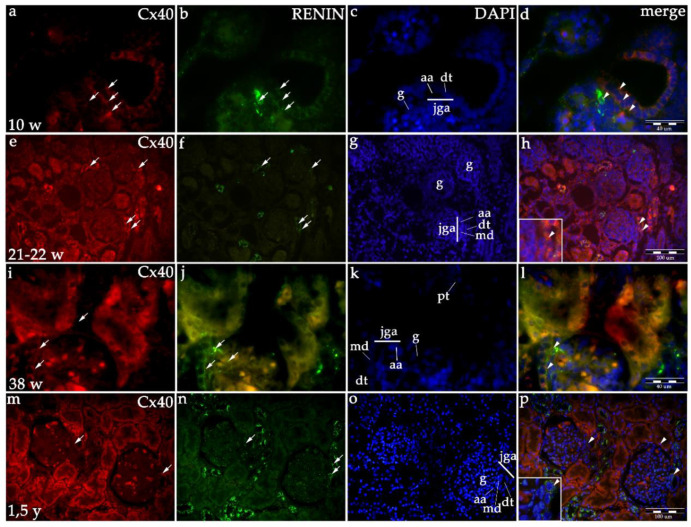
Co-expression of Cx40 and renin in the JGA region of developing and healthy postnatal human kidneys: kidneys in the 8th developmental week (**a**–**d**); kidneys in the 21–22nd developmental week (**e**–**h**); kidneys in the 38th developmental week (**i**–**l**), healthy postnatal human kidneys (**m**–**p**). Glomeruli (g), afferent arteriole (aa), macula densa (md), juxtaglomerular apparatus (jga), proximal tubule (pt), distal tubule (dt). Cxs or renin expression (arrows), DAPI nuclear stain (**c**,**g**,**k**,**o**). Merged microphotographs of different Cxs with renin (merge) show their co-expression (arrowheads) in the JGA (**d**,**h**,**l**,**p**). Double immunofluorescence staining of DAPI nuclear stain with Cx40/renin magnification ×40, scale bar 100 μm (**h**,**p**), ×100, scale bar 40 μm (**d**,**l**).

In the developing human kidneys, moderate (afferent arteriole) to strong expression (distal tubule) of Cx40 characterizes human JGA area. A gradual decrease in Cx40 expression in the JGA region is observed during further development, as well as in healthy postnatal kidneys (Table 1, Figure 1 and Figure 2). Linear model, R^2^ = 97.82% *p* = 0.01 (Figure 1).

#### 2.1.2. Cx43 Expression

**Figure 3:**
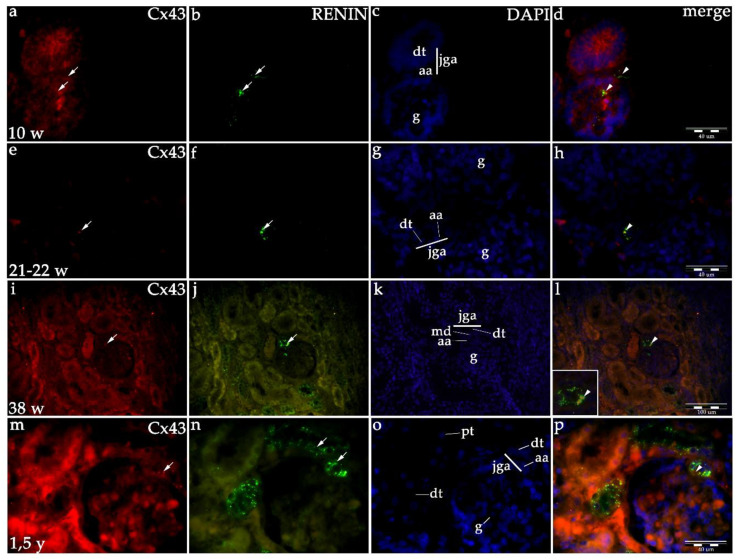
Co-expression of Cx43 and renin in the JGA region of developing and healthy postnatal human kidneys: kidneys in the 8th developmental week (**a**–**d**); kidneys in the 21–22th developmental week (**e**–**h**); kidneys in the 38th developmental week (**i**–**l**), healthy postnatal human kidneys (**m–p**). Glomeruli (g), afferent arteriole (aa), macula densa (md), juxtaglomerular apparatus (jga), proximal tubule (pt), distal tubule (dt). Cxs or renin expression (arrows), DAPI nuclear stain (**c**,**g**,**k**,**o**). Merged microphotographs of different Cxs with renin (merge) show their co-expression (arrowheads) in the jga (**d**,**h**,**l**,**p**). Double immunofluorescence staining of DAPI nuclear stain with Cx43/renin, magnification ×40, scale bar 100 μm (**l**), ×100, scale bar 40 μm (**d**,**h**,**p**).

Initially, Cx43 shows mild (afferent arteriole) to moderate (distal tubule) expression in developing JGA of early human kidneys. During further development Cx43 expression does not change in the JGA region until the 38th week, while in the late prenatal period and particularly in postnatal kidneys, it rises to moderate in afferent arteriole and strong in macula densa (Table 1, Figure 1 and Figure 3). Overall, any consistent trend could not be captured by linear modeling of Cx43 expression throughout studied developmental periods (R^2^ = 89.09% *p* = 0.0561, Figure 1).

#### 2.1.3. Cx45 Expression

**Figure 4:**
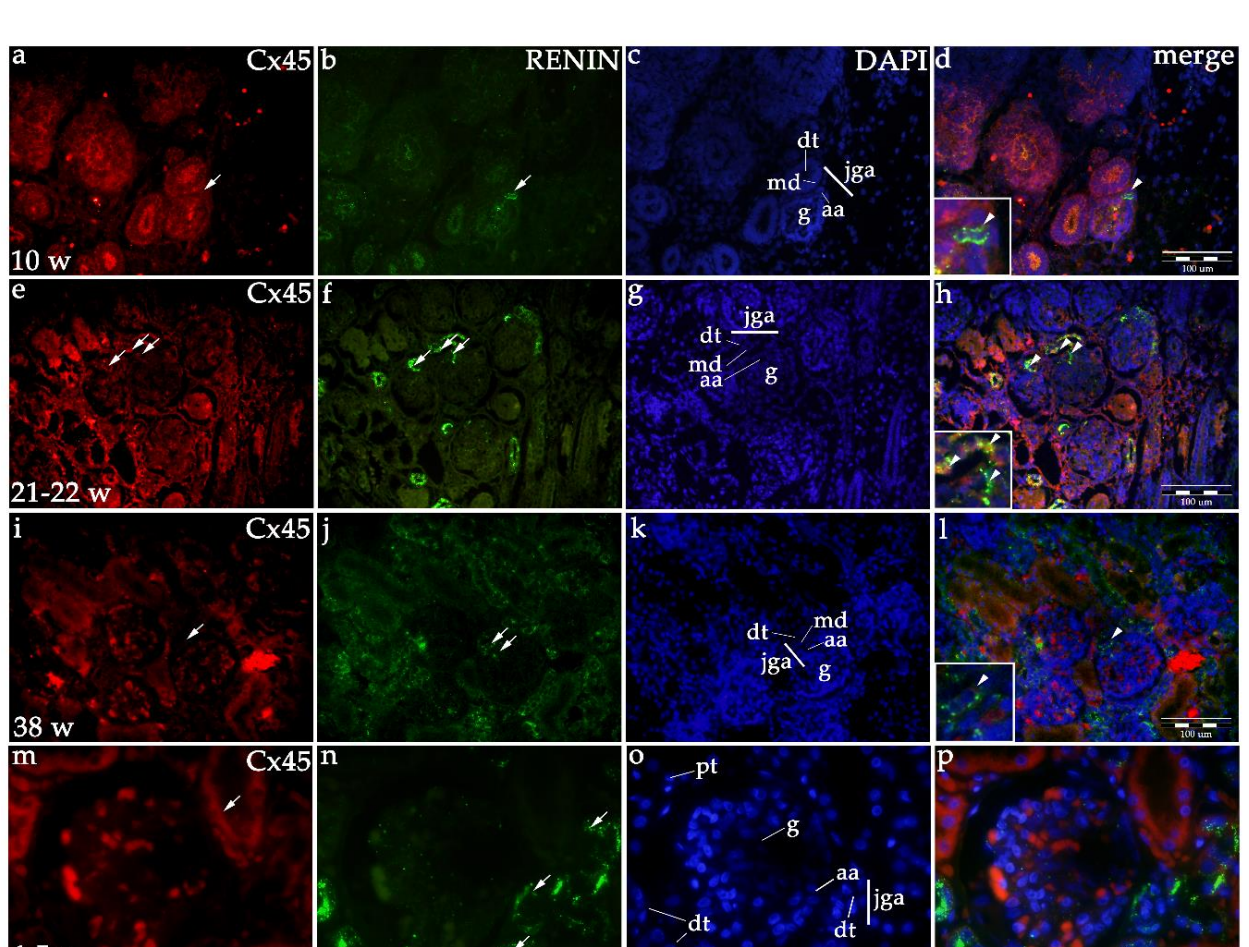
Co-expression of Cx45 and renin in the JGA region of developing and healthy postnatal human kidneys: kidneys in the 8th developmental week (**a**–**d**); kidneys in the 21–22th developmental week (**e**–**h**); kidneys in the 38th developmental week (**i**–**l**), healthy postnatal human kidneys (**m**–**p**). Glomeruli (g), afferent arteriole (aa), macula densa (md), juxtaglomerular apparatus (jga), proximal tubule (pt), distal tubule (dt). Cxs or renin expression (arrows), DAPI nuclear stain (**c**,**g**,**k**,**o**). Merged microphotographs of different Cxs with renin (merge) show their co-expression (arrowheads) in the jga (**d**,**h**,**l**,**p**). Double immunofluorescence staining of DAPI nuclear stain with Cx45/renin, magnification ×40, scale bar 100 μm (**d**,**h**,**l**), ×100, scale bar 40 μm (**p**).

Starting with the embryonic period (8th–10th developmental week) and continuing into the fetal period, Cx45 expression is mild (afferent arteriole) to moderate (distal tubule) in the JGA region. Afterward, Cx45 expression further rises to moderate/strong in the JGA region (Table 1, Figure 1 and Figure 4), but particularly in the proximal tubules which are not connected to JGA. If these observations for JGA is modelled with a line, a steady increasing linear trend can be noticed (R^2^ = 93.56% *p* = 0.0328, Figure 1).

#### 2.1.4. Cx37 Expression

**Figure 5:**
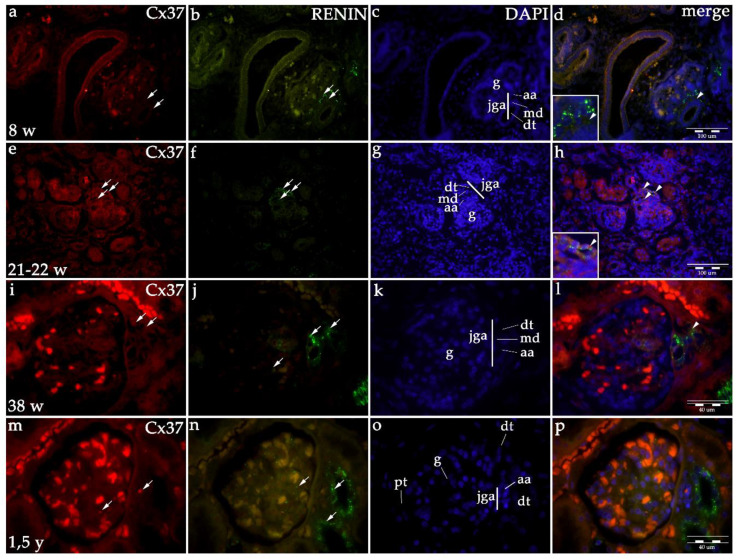
Co-expression of Cx37 and renin in the JGA region of developing and healthy postnatal human kidneys: kidneys in the 8th developmental week (**a**–**d**); kidneys in the 21–22th developmental week (**e**–**h**); kidneys in the 38th developmental week (**i**–**l**), healthy postnatal human kidneys (**m**–**p**). Glomeruli (g), afferent arteriole (aa), macula densa (md), juxtaglomerular apparatus (jga), proximal tubule (pt), distal tubule (dt). Cxs or renin expression (arrows), DAPI nuclear stain (**c**,**g**,**k**,**o**). Merged microphotographs of different Cxs with renin (merge) show their co-expression (arrowheads) in the jga (**d**,**h**,**l**,**p**). Double immunofluorescence staining of DAPI nuclear stain with Cx37/renin, magnification ×40, scale bar 100 μm (**d**,**h**), ×100, scale bar 40 μm (**l**,**p**).

In the 8th developmental week, mild Cx37 expression is observed in collecting tubules but not in the JGA region of immature glomeruli. From the 10th week on, Cx37 expression increases in the JGA region, primarily corresponding to distal tubules (macula densa) and afferent arteriole. In the postnatal period, Cx37 expression increases to moderate in the JGA and associated glomeruli (Table 1, Figure 1 and Figure 5). When formally tested for linear trend among developmental periods, the analysis did not show significance (R^2^ = 56.95% *p* = 0.2454, Figure 1).

Throughout development, expression of Cx40 is higher than the expression of Cx37, Cx43 and Cx45. In the postnatal period, expression of Cx43 decreases, while that of other Cxs increases (Figure 1).

#### 2.1.5. Co-Expression of Renin and Cxs

Co-expression of renin and Cxs are presented as overlapping areas of specific Cx and renin expression in the JGA region following the merging of captured images. By the end of the fetal period, co-expression is strong in the JGA region for Cx40 and Cx43, while it is less extensive and strong for the Cx45 and Cx37 (Figure 2, Figure 3, Figure 4 and Figure 5).

#### 2.1.6. Renin Expression in Developing and Postnatal Human Kidneys

**Figure 6:**
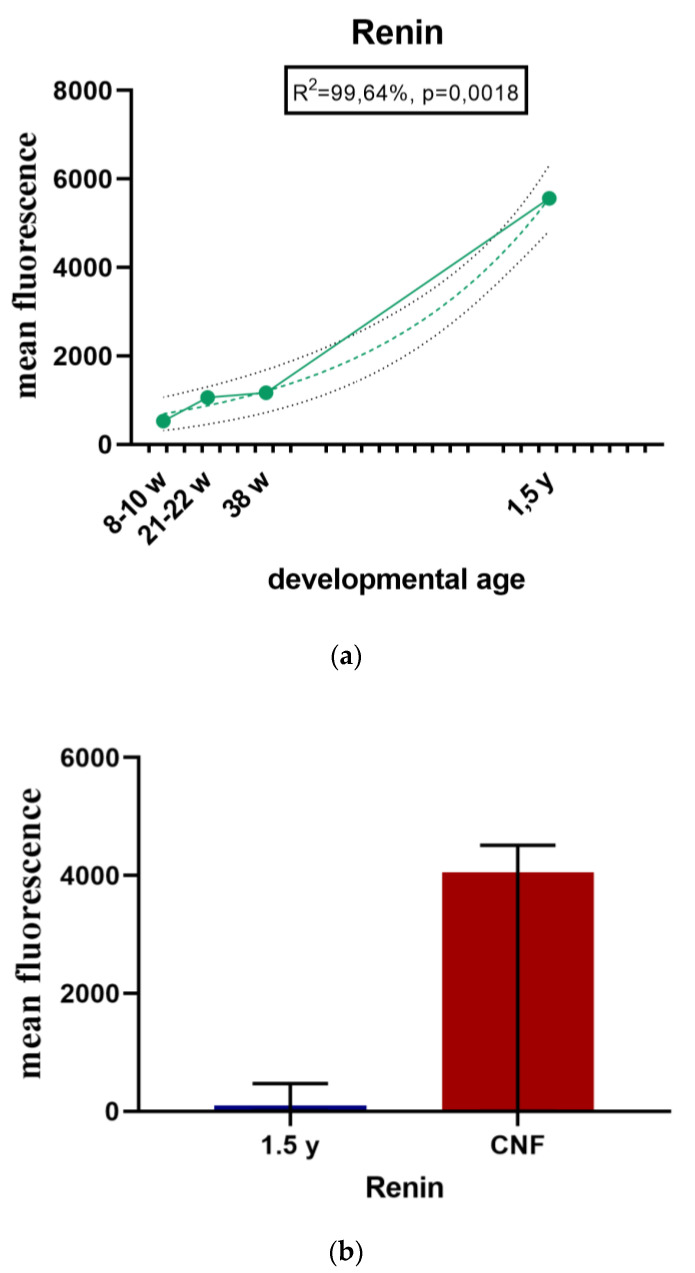
(**a**) Renin expression in the JGA region of developing and postnatal human kidneys shown by scatter plot; (**b**) Renin expression in the JGA region of healthy and CNF postnatal kidneys (median ± interquartile range for technical replicates).

During the early human development (8th–10th developmental week), renin-positive cells are observed in the developing kidney tissue, with the greatest accumulation of renin in the JGA regions of immature glomeruli. Throughout development, renin-positive cells are observed in afferent arteriole, but particularly dense renin granules characterize macula densa and part of distal tubules close to the JGA area of glomeruli. Low accumulations of renin granules are also observed inside the developing glomeruli and only occasionally in the interstitium (mesenchymal cells). In postnatal period, renin granules are predominantly observed in the distal tubules close to macula densa, in the macula densa and in the wall of afferent arteriole (see Figure 2, Figure 3, Figure 4 and Figure 5 and Figure 6a) but not in other parts of nephron and in the interstitium. Overall expression of renin in JGA showed exponential growth with the developmental age (R^2^ = 99.64%, *p* = 0.0018) (Figure 6a).

#### 2.1.7. Renin Expression in CNF Kidneys

In CNF kidneys, expression of renin significantly increases compared to healthy postnatal kidneys (Figure 6b).

### 2.2. CNF

In CNF kidneys, glomeruli are partly of normal size, but mostly smaller than normal. Some of them appear larger and lobulated, with an extensive proliferation of mesangial cells and segmental or global sclerosis.

**Figure 7:**
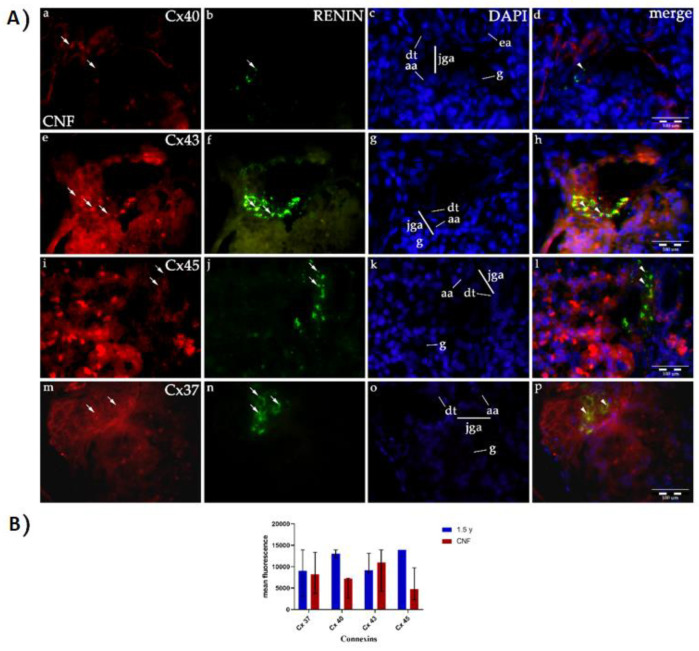
(**A**) Expression of different Cxs (Cx37, Cx40, Cx43, Cx45) and renin in the JGA region of CNF kidneys: glomeruli (g), afferent arteriole (aa), efferent arteriole (ea), distal tubule (dt), juxtaglomerular apparatus (jga), expression of Cxs or renin (arrows), co-expression of Cxs and renin (arrowheads). While expression of Cx40 is decreased in jga of CNF kidneys (**a**–**d**), expression of Cx43 (**e**–**h**), Cx45 (**i**–**l**) and Cx37 is increased (**m**–**p**). Double immunofluorescence staining of DAPI nuclear stain with Cx40/renin, Cx43/renin, Cx45/renin and Cx37/renin of CNF human kidneys magnification ×100, scale bar 40 μm. (**B**) Bar plot (median ± interquartile range for technical replicates) showing the difference between the expression of Cx37, Cx40, Cx43 and Cx45 in healthy postnatal and CNF kidneys.

Higher magnification of JGA region in CNF kidneys reveals expression of Cx40 in the macula densa and afferent arteriole, but also in glomeruli and parietal epithelial cells (Bowman’s capsule). Cx40 and renin granules co-express primarily in the JG cells of the afferent arteriole (Figure 7Aa–d).

In hypertrophic CNF glomeruli, Cx43 is strongly expressed in the afferent arteriole and macula densa of JGA. Cx43 and renin are strongly co-expressed in the JGA region, but also in glomeruli and cells corresponding to extraglomerular mesangium (Figure 7Ae–h).

In contrast to healthy postnatal JGA, strong expression of Cx45 characterizes macula densa and afferent arteriole of CNF kidneys. Increased expression of Cx45 is observed inside the glomerular cell population as well (Figure 7Ai–l).

Similar to Cx45, an increase in expression of Cx37 is observed in macula densa, afferent arteriole and in some glomerular and extraglomerular mesangial cells of CNF kidneys (Figure 7Am–p).

Compared to healthy kidneys, in CNF kidneys we observe reduced expression of Cx40, and increased expression of Cx43, Cx45 and Cx37 in the form of coarse granules in the JGA region of affected glomeruli (Figure 7B). However, among different glomeruli in CNF kidneys, we mostly analyzed large and lobulated ones, which also showed increased accumulations of renin in the JGA region (Figure 7A.). In contrast, small and sclerotic glomeruli did not show the presence of renin granules in the JGA region.

### 2.3. Semi-Thin Sections and Electron Microscopy of JGA in Healthy and CNF Postnatal Kidneys

**Figure 8:**
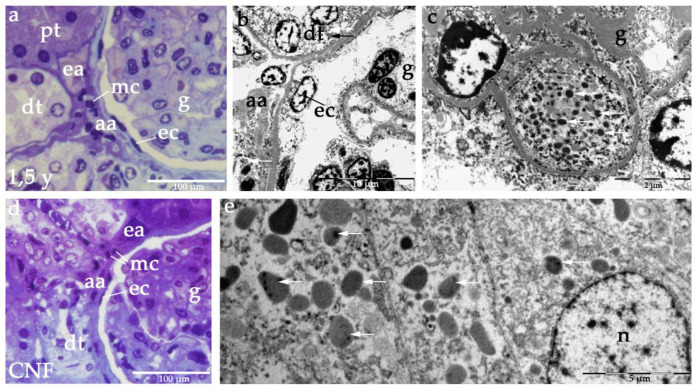
Toluidine blue staining semi-thin section (**a**) and electron microscopy of JGA region of heathy postnatal kidneys (**b**) shows characteristic JGA structures: distal tubule (dt), proximal tubule (pt), afferent arteriole (aa), efferent arteriole (ea), extraglomerular mesangial cells (mc), glomeruli (g), parietal epithelial cells (ec). Note renin granules (arrows) in dt and aa, as well as in some glomerular cells (**c**). Toluidine blue staining semi thin section of JGA region in CNF (**d**) is showing the same histological structures. Electron microscopy shows dense accumulations of renin granules in macula densa of CNF kidneys (**e**). Scale bars: 100 μm (**a**,**d**), 10 μm (**b**), 2 μm (**c**), 5 μm (**e**).

The JGA region of healthy postnatal kidneys contains a section through afferent and efferent arteriole, distal tubule with macula densa and extraglomerular mesangial cells (Figure 8a). In electron microscopy, renin granules are observed in afferent arteriole and distal tubules (Figure 8b), while some renin containing cells can be found inside the glomeruli as well (Figure 8c).

In CNF kidneys, the JGA region contains the same constitutive structures, while mesangial cells appear more numerous (Figure 8d). In distal tubules of CNF kidneys, greater accumulations of renin granules are observed (Figure 8e).

## 3. Discussion

Our study has shown that already in the 8th developmental week the kidney tissue starts organizing the JGA region by the closely apposed juxtaglomerular cells in afferent arteriole, cells in the distal tubule (macula densa) and the extraglomerular mesangium. At the end of the embryonic period, different Cxs can be observed within the JGA structures, showing particularly strong Cx40 expression, while Cx37, Cx43 and Cx45 display less intense expression patterns. During further development, between the mid-gestation and prenatal period, Cx40 decreases while other Cxs gradually increase to finally reach more equal intensity levels in the postnatal period. These findings indicate the importance of different Cxs, but particularly Cx40 in the formation of prenatal JGA. In our study, the process of JGA formation was associated with increased renin accumulation, showing its peak in the postnatal period, when the newborn child establishes its own blood pressure control system. Both renin and connexins have been previously shown to participate in maintaining kidney homeostasis, as well as in responding to changes in normal kidney functions [6,47,48]. However, there has been conflicting evidence on their expression pattern, particularly during development. In addition, we have found that the expression of Cxs in JGA had its own characteristic pattern when compared to other parts of the kidney tissue [49]. Renin positive cells, which were dispersed in the kidney tissue during development, later on concentrated in the JGA, which implies their possible role in several aspects of human development. Similar observations have been made in earlier studies on human tissue, as well as in animal experiments [9,20]. In rats, renin mRNA has been found in the vascular pole of juxtamedullary glomeruli and along afferent, interlobular, and arcuate arteries [50]. In our study, besides the presence of renin granules in JC and temporarily in mesenchymal (interstitial) cells of developing kidneys, we also found renin granules in distal convoluted tubules, which other studies have failed to show. In a study by Minuth et al. on fetuses and newborn mice, renin was detected in the media of interlobular kidney arteries even prior to renin appearance in other organs [20]. In contrast, we observed renin preferentially in the JGA area already during early embryonic development. However, both studies showed that the concentration of renin increased rapidly after birth. Schutz et al. investigated expression of different RAS components during the earliest embryonic and fetal stages of human development and reported renin expression in the JGA and arteries closest to the glomeruli. While those authors observed renin mRNA in other organs such as the chorion and heart, in contrast to our study they did not detect the presence of renin granules in the distal tubules, including macula densa [20,51].

In CNF kidneys, in contrast to other Cxs which showed increased expression in the JGA region, we found decreased levels of Cx40 compared to healthy postnatal kidneys. The highest expression intensity characterized Cx43, followed by Cx37 and Cx45. Considering increased accumulations of renin in hypertrophic glomeruli of CNF kidneys, we speculate that it might be due to the process by which the function of sclerotic glomeruli, which are devoid of renin expression, is compensated. We also found renin and associated Cx expression in the neighboring extraglomerular mesangial cells in the JGA of CNF kidneys, thus implying that reappearance of the embryonic pattern of their expression is possible in pathological conditions such as CKD caused by CNF. This can be explained by the ability of those cells to produce renin in states of threatened homeostasis, which has been already earlier addressed [10]. Studies on animals pointed to increased renin synthesis and release in various situations, such as low systemic blood pressure, hypovolemia, sodium deprivation, and sympathetic stimulation [16]. In addition, recent animal studies suggested an important role of RAS activation during and after injury in the AKI-CKD continuum [24]. RAS has been considered as an important part of other renal fibrotic/hypertrophic diseases as well [21,52]. Similar to our results, a study on human kidneys has shown evidence of Cx37, Cx40 and Cx43 expression in the extraglomerular mesangium of JGA [53]. In contrast, studies on developing and adult mice showed only Cx40-renin co-expression [43], while the displacement of renin-producing cells to extraglomerular mesangium was observed only in the absence of Cx40 [34]. In addition, in mice lacking Cx40, renin-producing cells were disorganized and dysfunctional [54,55], while in Cx37 null mice investigators have failed to show any changes in renin positioning and activity [42]. Results similar to Cx37 expression were found regarding Cx43 in mice [56], while opposite results were presented in the study on hypertensive mice which indicated the importance of Cx43 in renin secretion [57]. These discrepancies in results could be explained by inter-species differences or methodology used in the particular study. Namely, comparison of data analyzing mouse and human development often displays some interspecies differences which appear during the progression of development, while more sophisticated methods can make easier earlier detection of some markers in developing tissues.

Our findings support the important role of Cx40 in renin-producing cells [43,44], but also the role of the other three Cxs in pathological kidney conditions such as CNF. It has been speculated earlier that Cx40 could be replaced by Cx45, which has also been shown as slightly increased in our study of CNF kidneys [45]. We suggest that while Cx40 has a major role in JGA formation during development, other Cxs take over its role in the postnatal period. We also noticed an association in the increase of Cxs expression with greater accumulation of renin not only in JG cells but also in macula densa cells and extraglomerular mesangium. An earlier study suggested that renin granules are released by paracrine secretion associated with shrinkage of macula densa cells [12]. This can be another explanation for the increased presence of renin granules in the area populated by mesangial cells, which we observed in the affected hypertrophic glomeruli of CNF kidneys.

In conclusion, our study has shown the expression of different Cxs and parallel appearance of renin granules in the JGA region already during early human kidney development. Based on their expression pattern, Cx40 seems to have a key role in the development of JGA, while Cx37, Cx43 and Cx45 take over its role in the postnatal period. Dispersed renin cells gradually reduce their localization to the JGA region, with increasingly accumulated granules in the postnatal period. Compared to healthy postnatal kidneys, CNF kidney tissue displays increased expression of Cx37, Cx43 and Cx45 in the JGA region of hypertrophic glomeruli, while Cx40 expression becomes weaker. Renin granules in JGA additionally spread to extraglomerular mesangial cells in CNF kidneys, thus reestablishing their embryonic expression pattern. The analyzed co-expression of Cxs and renin cells in developing, healthy and CNF human kidneys indicates that Cx40 primarily controls JGA formation, while Cx37, Cx43 and Cx45 might be involved in signal transfer within the JGA region and postnatal maintenance of kidney function equilibrium, including blood pressure control.

## 4. Materials and Methods

### 4.1. Human Material

A total of 10 tissue samples of developing kidney tissue were obtained from the Department of Gynecology and Obstetrics and the Department of Pathology with permission of the Ethical and Drug Committee of the University Hospital in Split in accordance with the Helsinki Declaration (class: 003-08/16-03/0001, approval number: 2181-198-03-04-16-0024). Embryonic and fetal tissues were acquired after spontaneous abortions or after tubal pregnancies, and only morphologically regular conceptuses without signs of macerations were used in our study. The age of conceptuses was evaluated between 8th and 38th developmental week from menstrual data corresponding to the external measurements (crown–rump length) [58]. Postnatal tissue used in our study was collected during the autopsy of healthy 1, 1.5 and 7-years old children and from 3 nephrectomized CNF patients (homozygous missense mutation c.1096 A > C; pSer366Arg in *NPHS1*, the gene was detected in all three patients). For that part of the conducted research, we obtained informed parental consent.

### 4.2. Immunohistochemistry and Immunofluorescence Staining

Tissue samples of caudal parts containing kidneys were dissected, fixed in 4% paraformaldehyde in phosphate buffer and dehydrated in 100% ethanol. Tissues were processed as we previously described [1,59]. It was paraffin-embedded and serially cut as 5 µm thick sections. To confirm tissue preservation, we used HE staining of every 10th section.

Preparation for immunofluorescence followed standard protocol [60]. Deparaffinisation and tissue rehydration was followed by heating in a sodium citrate buffer for 20 min at 95 °C. After washing the tissue with PBS, tissue was covered with a blocking buffer (Protein Block ab64226, Abcam, Cambridge, UK) to prevent non-specific staining. The samples were incubated with primary antibodies (Table 2) overnight and again rinsed in PBS. Secondary antibodies were then applied (Table 2) and incubated in a humidity chamber for one hour. Subsequently, slides were washed in PBS, nuclei were counterstained with DAPI and slides were air-dried and cover-slipped (Immuno-Mount, Thermo Shandon, Pittsburgh, PA, USA) [1]. To prove specificity, we used control slides for which we excluded primary antibodies from the staining procedure.

A BX61 fluorescence microscope (Olympus, Tokyo, Japan) equipped with a digital camera (DP71) was used for imaging. Images were captured using the Olympus CellA software and plates were assembled using Adobe Photoshop.

### 4.3. Semi-Thin and Ultra-Thin Sections and Electron Microscopy

Tissue samples of 10th and 22nd week-old human kidneys, 1.5 years healthy and 3-year CNF kidney tissue were used in the preparation of slides for electron microscopy. After the 24-hours fixation in 4% paraformaldehyde, specimens were post-fixed in 1% osmium tetroxide for an hour, followed by dehydration in courses of ethanol. Samples were embedded in LX 112 resin. Previously cut semi-thin sections (1 µm) were stained using the toluidine blue. Semi-thin sections were sliced into ultra-thin sections (0.05 µm thick), stained with uranyl acetate and lead citrate and lastly examined by transmission electron microscope (Zeiss 902 A, Jena, Germany) [1].

### 4.4. Semi-Quantification

The staining intensity of chosen antibodies was estimated using four categories: absence of any reactivity (−); mild reactivity (+); moderate reactivity (++) and strong reactivity (+++). Semi-quantification was performed by three autonomous researchers using the image analysis software ImageJ (National Institutes of Health (NIH), Bethesda, MD, USA) (Table 1).

### 4.5. Statistics and Microphotograph Quantification

Fluorescence intensity histograms were acquired for red and green fluorescence channels in ImageJ software (NIH). The threshold for background exclusion was set by three experienced histologists. Expression of different proteins was quantified as the area under the curve (AUC) of fluorescence intensity histograms, and we refer to this as mean fluorescence in the rest of the article. AUCs and their interval estimates were calculated by using the AUC analysis routine in GraphPad Prism 8.0 software (Graph Pad, La Jolla, CA, USA).

For studying expression dynamics and trends of different proteins through developmental periods linear and nonlinear regression modeling was used in GraphPad Prism 8.3 software. Confidence in models is presented as 95% CI on graphs. Comparisons in mean fluorescence between two groups were done with Mann-Whitney using the same software. The level of significance was set at *p* = 0.05.

## Figures and Tables

**Table 1:** Expression of different Cxs in the JGA of developing kidneys, postnatal healthy and CNF kidneys.

Weeks/Years	Cx40	Cx43	Cx45	Cx37
8–10 weeks	++/+++	+/++	+/++	+/++
21–22 weeks	++/+++	+/++	+/++	+/++
38 weeks	++	+/++	+/++	+/++
1.5 year	+/++	++/+++	++	++
CNF—cca 1.5 year	+	+++	++/+++	++/+++

**Table 2:** Primary and secondary antibodies used in the study.

	Antibodies	Host	Dilution	Source
Primary	Anti-Cx37/GJA4 ab181701	Rabbit	1:500	Abcam (Cambridge, UK)
	Anti-Cx40/GJA5 ab213688	Rabbit	1:100	Abcam (Cambridge, UK)
	Anti-Cx43&GJA1 ab87645	Goat	1:200	Abcam (Cambridge, UK)
	Anti-Cx45/GJA7 ab135474	Rabbit	1:100	Abcam (Cambridge, UK)
	Anti-Renin [7D3-E3] (ab134783)	Mouse	1:100	Abcam (Cambridge, UK)
Secondary	Anti-Goat IgG, Rhodamine Red™-X (RRX) 705-295-003	Donkey	1:400	Jackson Immuno Research Laboratories, Inc., Baltimore, PA, USA
	Anti-Rabbit IgG, Rhodamine (TRITC) 711-025-152	Donkey	1:400	Jackson Immuno Research Laboratories, Inc., Baltimore, PA, USA
	Anti-Mouse IgG, Alexa Fluor^®^ 488 ab150105	Donkey	1:400	Abcam (Cambridge, UK)

## Abbreviations

MET	Mesenchymal to epithelial transition
JC	Juxtaglomerular cells
JGA	Juxtaglomerular apparatus
EM	Extraglomerular mesangium
VSMCs	Vascular smooth muscle cells
RAS	Renin-angiotensin-aldosterone system
AKI-CKD	Acute kidney injury to chronic kidney disease transition
Cxs	Connexins
CKD	Chronic kidney disease
CNF	Nephrotic syndrome of the Finnish type
